# A Timely Reminder

**Published:** 1902-08

**Authors:** 


### A TIMELY REMINDER.

Dr. George M. Gould, of Philadelphia, reports six cases of epilepsy due to ametropic eye strain, all of which were promptly cured by the application of suitable glasses.

It is worthy of note that the eyes of several of these had been examined on previous occasions without a midriatic, and that glasses prescribed at these times had not afforded any relief.

Such well-authenticated reports serve to remind us that unsuspected eye strain is often the chief causative factor in nervous diseases of obscure origin, and serve to emphasize the need for having all eye examinations carefully made, and made by competent men only. As a rule, general practitioners attach far too little importance to the detection of ametropia and its correction. Many otherwise careful and intelligent men seem not to realize the unwisdom of trusting their eye cases to the average optician or to an inexperienced specialist. It is strange that this should be true, since every physician understands that an unskillfully done refraction, followed by the application of improperly ground lenses, injures the patient *directly* by increasing the existing error, and, *indirectly*, may do infinite harm by misleading the attending physician into seeking elsewhere for the seat of trouble. R.

